# Reply: Correcting Misaligned Risk Comparisons Between LDL-C Levels and Polygenic Risk Scores

**DOI:** 10.1016/j.jacadv.2026.102685

**Published:** 2026-03-19

**Authors:** Carlos Iribarren, Roberto Elosua, Martha Gulati, Nathan D. Wong, Jamal S. Rana

**Affiliations:** a Kaiser Permanente Northern California Division of Research, Pleasanton, California, USA; b Institut Hospital del Mar d’Investigacions Mèdiques (IMIM), Barcelona, Spain; c Spain and CIBER Cardiovascular Diseases (CIBERCV), Barcelona, Spain; d University of Vic-Central University of Catalonia (UVic-UCC), Vic, Spain; e Davis Women's Heart Center, Houston Methodist DeBakey Heart & Vascular Center, Houston, Texas, USA; f University of California-Irvine, Irvine, California, USA; g The Permanente Medical Group, Kaiser Permanente Oakland Medical Center, Oakland, California, USA

We thank Drs Botta and Domenico for their commentary on our 2025 Brief Communication in *JACC: Advances* examining the joint effect of low density lipoprotein cholesterol and polygenic risk on coronary heart disease risk in the GERA cohort.^1^

Botta and Domenico recognize the methodological differences between the UK Biobank^2^ and genetic epidemiology research on aging (GERA)^1^ studies that led to seemingly different results. However, it is reassuring that when they reanalyzed the UK Biobank data with our approach using low polygenic risk score (PRS)∖low LDL (<100 mg/dL) as the reference group, a remarkably similar pattern emerged between the 2 studies. In particular, it is important to point out the significant effect of very high LDL (>190 mg/dL)/low PRS vs low LDL/low PRS (HR: 2.21; 95% CI: 1.21-4.04). Unlike GERA (where there was an effect), their original approach did not find an effect of very high LDL in the low PRS group. Whereas it is true that, with the same analytical approach as ours in the GERA cohort, their PRS (with ∼300K [single nucleotide polymorphisms]) yielded stronger associations than our PRS with 12 single nucleotide polymorphisms (CARDIO inCode-Score), there remain differences between studies that ought to be considered to put these findings into perspective. First, Botta and Domenico used the upper and lower deciles of the PRS distribution to define low and high PRS, respectively, while we used the upper and lower quintiles. In addition, they excluded individuals on antihypertensive medication, whereas in GERA, 36% of the cohort had either diagnosis or treatment for hypertension. If this exclusion is not uniform across PRS groups, it can lead to distortion of risk estimates.

Regarding the number of genetic variants included in our PRS, our rationale was to incorporate variants independently associated with coronary heart disease and not related to classical cardiovascular risk factors, with the aim of improving the predictive performance of cardiovascular risk functions. In a previous study in which we analyzed a PRS comprising more than 50 variants, the results were comparable to those obtained with the 12-variant PRS.^3^ Similarly, Truong et al reported comparable effect sizes and predictive performance when using a genome-wide PRS and a more restricted PRS (n = 267 variants).^4^ In this context, there is an ongoing debate regarding the optimal strategy for constructing PRSs, which extends beyond the mere number of variants included.^5^

The take-home message remains unchanged: polygenic risk modulates the risk threshold for elevated low density lipoprotein cholesterol, and more aggressive primary prevention is warranted for individuals with high polygenic risk.
